# Effectiveness and safety of osimertinib in patients with metastatic EGFR T790M-positive NSCLC: An observational real-world study

**DOI:** 10.1371/journal.pone.0221575

**Published:** 2019-08-23

**Authors:** Yabing Cao, Xibin Qiu, Guangli Xiao, Hao Hu, Tongyu Lin

**Affiliations:** 1 Department of Oncology, Kiang Wu Hospital, Macau, China; 2 Department of Medical Oncology, Sun Yat-Sen University Cancer Center, Guangzhou, China; Istituto di Ricovero e Cura a Carattere Scientifico Centro di Riferimento Oncologico della Basilicata, ITALY

## Abstract

Osimertinib showed encouraging efficacy in patients with advanced EGFR T790M-positive NSCLC in previous randomized controlled trials. This real-world study aimed to evaluate the effectiveness and safety of osimertinib in a real-world setting. This observational study (NCT03133234) included 74 patients with metastatic EGFR T790M-positive NSCLC who progressed on prior EGFR TKI therapy and received osimertinib between May 2016 and April 2018 at the Kiang Wu Hospital in Macau. Response rate (RR) and other endpoints (progression-free survival [PFS], overall survival [OS], disease control rate [DCR], stable disease rate, and adverse events) were assessed. Survival data were estimated using the Kaplan-Meier method. All patients had stage IV lung adenocarcinoma and 25.6% had brain metastases; median age was 58 years (range 28–84 years) and 83.8% of patients had received at least three prior lines of treatment. The median duration of osimertinib treatment was 8 months (range, 1–25 months). RR and DCR were 67.5% (95% CI 56.9–78.1) and 79.8% (95% CI 70.7–88.9), respectively, while 12.1% had stable disease. The median PFS was 9.0 months (95% CI 6.7–11.2 months), and the median OS was 12.0 months (95% CI 8.8–15.1 months). Nausea (25.8%) and decreased appetite (20.2%) were the most common adverse events associated with osimertinib treatment. Even though most patients had at least three lines of prior treatment, real-world RR and PFS with osimertinib in this study were consistent with those from randomized controlled trials; no new safety signals were observed.

## Introduction

Mutations on the epidermal growth factor receptor (EGFR) genes are known to alter sensitivity of treatment in lung cancer [1]. The majority of EGFR tyrosine kinase domain mutations has been described as deletions in exon 19 or point mutations in exon 21 arising from substitution of leucine to arginine at codon 858 (L858R) [2].

Currently available first-line treatment for locally advanced or metastatic non-small cell lung cancer (NSCLC) harboring EGFR mutations include EGFR tyrosine kinase inhibitors (TKIs) such as gefitinib, erlotinib, afatinib, and more recently, osimertinib [3, 4]. Earlier studies report outstanding response rates with these TKIs, and median progression-free survival (PFS) ranging between 9 and 13 months [5–10]. However, most patients ultimately develop resistance to TKIs, resulting in disease progression; of which approximately half is due to EGFR T790M mutation [11].

EGFR T790M mutation—whereby threonine replaces methionine at position 790 of the EGFR gene domain in exon 20 –represents the major mechanism of acquired resistance, and usually arises as a result of long-term treatment [12]. Osimertinib is a third-generation, irreversible EGFR TKI that is selective for EGFR-activating and T790M resistance mutation, and is also able to penetrate the blood-brain barrier for activity in the central nervous system (CNS) [13].

Osimertinib was first granted approval by FDA in 2015 [14], two years after receiving accelerated approval, for treatment of patients with metastatic EGFR T790M-positive NSCLC who have progressed on or after EGFR TKI [14]. The initial approval of osimertinib for EGFR T790M-positive NSCLC was based on the results of the AURA3 trial [15], which demonstrated significantly longer median PFS with osimertinib than with platinum therapy plus pemetrexed (10.1 months vs. 4.4 months, respectively). In the same trial, the objective response rate was 71%, and the majority of patients (69%) had a partial response with 93% disease control rate (DCR) [15]. Safety results from the AURA3 trial demonstrated that osimertinib was generally well-tolerated, with a lower incidence of adverse events of grade 3 and above (23%) than its comparator (platinum therapy plus pemetrexed; 47%) [15].

Randomized controlled trials of osimertinib showed promising efficacy in patients with advanced EGFR T790M-positive NSCLC; however, further evaluation is needed in the real-world where the patient population is more diverse. Hence, this study aimed to evaluate the effectiveness and safety of osimertinib in Chinese patients with metastatic EGFR T790M-positive NSCLC in a real-world setting.

## Materials and methods

### Study design and patients

This observational study was conducted at the Kiang Wu Hospital in Macau SAR, China. This study was approved by the Institutional Review Boards of the Kiang Wu Hospital. Approval number: 2016–016. All patients voluntarily signed an informed consent form.” Patients who met the following eligibility criteria were enrolled consecutively. Inclusion criteria: age >18 years; locally advanced (stage IIIB) or metastatic (stage IV) NSCLC not amenable to curative surgery or radiotherapy; confirmed T790M mutation; disease progression on previous EGFR TKI treatment with or without additional lines of treatment; and treatment with osimertinib at the participating site between May 2016 and April 2018. According to study protocol, sample size of the study was estimated to be 50 patients. This sample size will be sufficient to estimate a median PFS of 10 months with 95% confidence interval of 7.2–13.9 months and allow a precision of ±13.6% around a point estimate for response rate of 60% in a real world setting. Exclusion criteria: enrollment in other studies that prohibit participation in this study. Enrolled patients received osimertinib 80 mg once daily until physician-assessed disease progression. Outcome measures were extracted from patient medical records. Recruitment date was from May 2016 to April 2018, and cut-off date of follow-up was July 28, 2018.

The local independent ethics committee reviewed and approved the study protocol and documents used for informed consent prior to study initiation. The protocol of this study can be found at http://dx.doi.org/10.17504/protocols.io.2qygdxw. All study procedures were conducted in accordance with the Declaration of Helsinki and in compliance with local regulatory requirements. All patients provided written informed consent before any study-related procedures were performed. This study is registered with ClinicalTrials.gov (identifier NCT03133234).

### Study endpoints and assessments

The primary efficacy endpoint was the response rate (RR), defined as the proportion of patients with complete response (CR) or partial response (PR) to treatment, as assessed by the physician. Other efficacy endpoints included PFS, OS, DCR (defined as the percentage of patients who had complete response, partial response, or stable disease) and the proportions of patients with stable disease or progressive disease. Treatment response and disease progression were assessed by computed tomography or positron emission tomography/computed tomography. We use RECIST 1.1 (investigator assessment) for efficacy evaluation. The PFS was calculated from start date of Osimertinib treatment. Safety endpoints included incidence of adverse events (including abnormal laboratory findings) of any grade and of grades 3 and above. In the event of severe adverse events (i.e. grades 3 and above), osimertinib dose was reduced. Osimertinib treatment was only resumed upon complete resolution of symptoms. Adverse events were graded according to the National Cancer Institute Common Terminology Criteria for Adverse Events version 4.0.

### Statistical analyses

Patient demographics, baseline disease characteristics, and adverse events were summarized using descriptive statistics. The Kaplan-Meier method was used to estimate the PFS and OS of the overall patient population, and the Cox proportional hazards model to estimate the 95% CIs. OS among patients with and without brain metastases was also analyzed. The data cut-off date was 28 July 2018. Statistical analyses were performed using the SPSS version 20.0 software package (SPSS Inc., Chicago, IL, USA).

## Results

### Patient demographics and baseline characteristics

**Fig 1:**
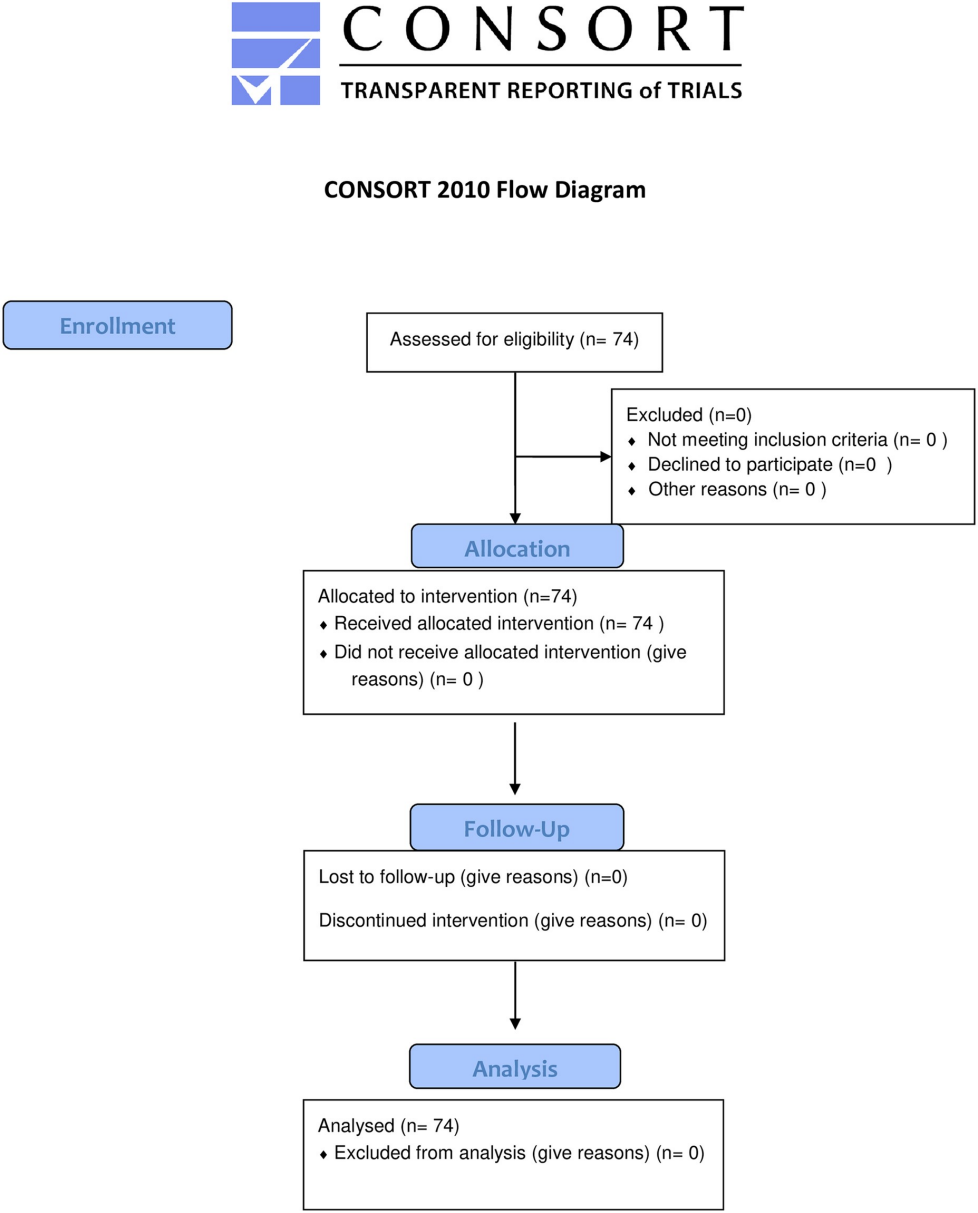
Flowchart of study design.

A total of 74 patients were enrolled, which was about 50% extended to the estimated sample size (Fig 1). All patients continued with treatment until disease progression as assessed by their physician; none discontinued the study early. At the date of data cut-off, eight patients were still receiving treatment. Demographics and baseline characteristics of these patients are shown in Table 1. All patients had stage IV lung adenocarcinoma, and 25.6% had brain metastases. The presence of EGFR T790M mutation was confirmed in all patients by polymerase chain reaction (64.8%) or next generation sequencing (35.1%). All patients were previously treated with TKI and 83.8% of patients had received at least three prior lines of treatment. The median duration of osimertinib treatment was 8 months (range, 1–25 months), and the median follow-up time was 9 months (range, 1–27 months).

**Table 1:** Patient demographics and baseline characteristics.

	All patients (N = 74)
**Age in years, median (range)**	58 (28–84)
**Gender**	
** Male**	25 (33.7)
** Female**	49 (66.3)
**Smoking history**	
**Ever**	8 (10.8)
**Never**	66 (90.2)
**Stage IV**	74 (100)
**Brain metastasis**	19 (25.6)
**Prior treatment**	
**Chemotherapy**	25 (33.7)
**EGFR TKI**	74 (100.0)
**Combination**	1 (1.3)
**Lines of treatment**	
** ≤2**	12 (16.2)
** 3**	38 (51.5)
** ≥4**	24 (32.3)
**WHO Performance Status**	
**0**	15 (20.3)
**1**	52 (70.3)
**2**	7 (9.5)
**T790M test method**	
**PCR**	48 (64.8)
**NGS**	26 (35.1)
**Sample type**	
**Blood**	16 (21.6)
**Tissue**	58 (78.3)

Values are presented as number (%) unless otherwise stated.

Percentages may not sum to exactly 100 due to rounding.

NGS, next generation sequencing; PCR, polymerase chain reaction; EGFR TKI, epidermal growth factor tyrosine kinase inhibitor; WHO, World Health Organization

### Efficacy

**Fig 2:**
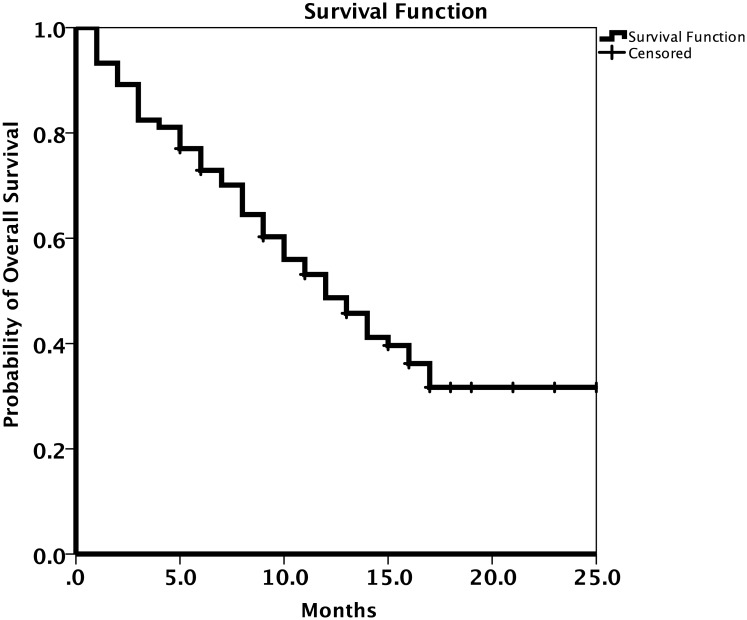
Kaplan-Meier estimates of progression free survival (PFS) with osimertinib treatment.

**Fig 3:**
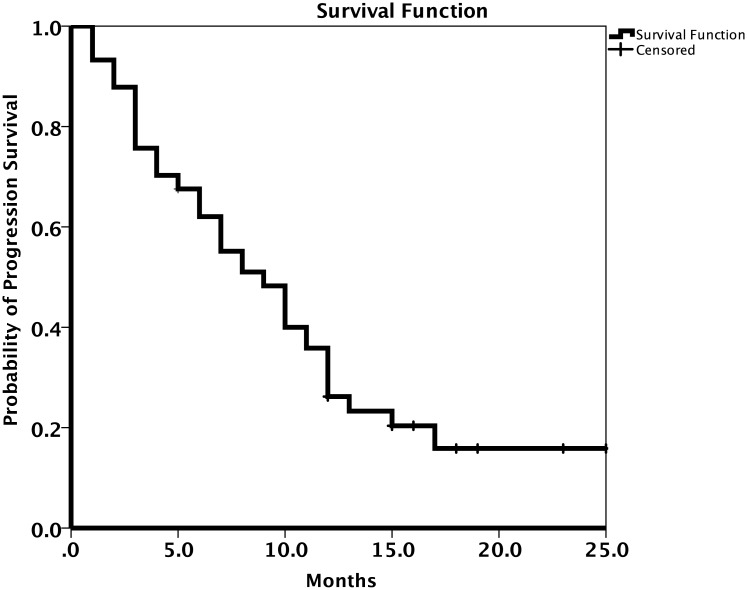
Kaplan-Meier estimates of overall survival (OS) with osimertinib treatment.

**Fig 4:**
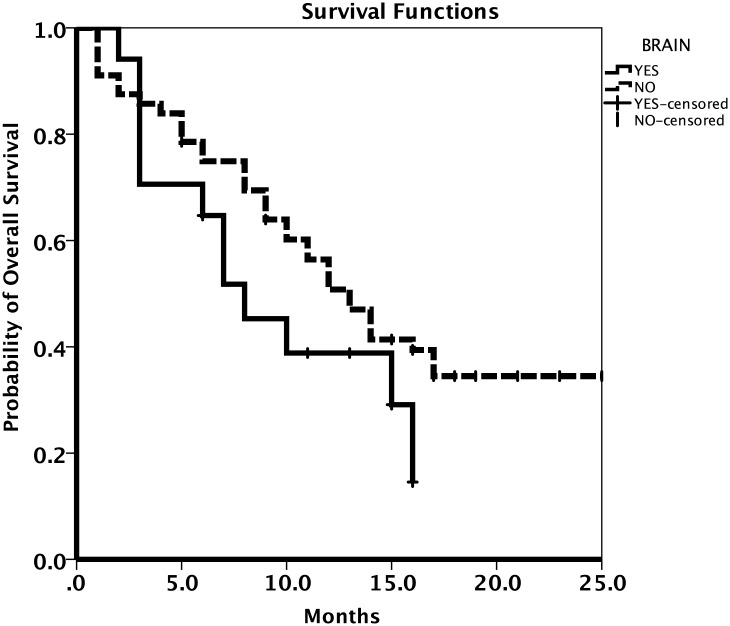
Kaplan-Meier estimates of overall survival for patients with or without brain metastases.

A summary of the patients’ response to treatment is provided in Table 2. Most patients (62.1%) had a partial response to osimertinib treatment. The RR and DCR were 67.5% (95% CI 56.9–78.1%) and 79.8% (95% CI 70.7–88.9%), respectively. The results for patient survival are presented in Figs 2 and 3. The median PFS was 9.0 months (95% CI 6.7–11.2 months; Fig 2) and the median OS was 12.0 months (95% CI 8.8–15.1 months; Fig 3). The median OS for patients with brain metastases was 8.0 months (95% CI 4.2–11.8 months) and that for patients without brain metastases was 13.0 months (95% CI 10.4–15.6 months; Fig 4).

**Table 2:** Response to osimertinib treatment.

Response	All patients (N = 74)	95% CI
Type of response		
CR	4 (5.4%)	-
PR	46 (62.1%)	-
SD	9 (12.1%)	-
PD^†^	15 (20.2%)	-
Response rate (CR + PR)	50 (67.5%)	56.9–78.1
Disease control rate (CR + PR + SD)	59 (79.8%)	70.7–88.9

Percentages may not sum to exactly 100 due to rounding.

^†^including death during treatment with or without relation to osimertinib

CI, confidence intervals; CR, complete response; PD, progressive disease; PR, partial response; SD, stable disease

### Safety

A summary of adverse events and laboratory abnormalities is provided in Table 3. Most adverse events were mild to moderate in severity (grades 1–2). The most common adverse events of any grade were nausea (45.9%), diarrhea (31.0%), and decreased appetite (28.3%). The most common adverse events related to osimertinib treatment were nausea (25.8%), decreased appetite (20.2%), and diarrhea (16.2%). Diarrhea was the most common adverse event of grades 3 and above, having occurred in 2.7% of patients. The most common laboratory abnormalities of any grade were alanine/aspartate aminotransferase elevation (25.6%), thrombocytopenia (17.5%), and neutropenia (16.2%). Neutropenia was the most common laboratory abnormality of grades 3 and above, having occurred in 2.7% of patients. Interstitial lung disease occurred in two patients (2.7%).

**Table 3:** Summary of adverse events and laboratory abnormalities (N = 74).

Adverse events^†^	Any Grade^§^	≥ Grade 3^§^
Nausea	34 (45.9)	1 (1.3)
Diarrhea	23 (31.0)	2 (2.7)
Decreased appetite	21 (28.3)	1 (1.3)
Fatigue	14 (18.9)	1 (1.3)
Rash	12 (16.2)	1 (1.3)
Constipation	11 (14.8)	0 (0.0)
Vomiting	8 (10.8)	0 (0.0)
Laboratory abnormalities		
ALT/AST elevation	19 (25.6)	1 (1.3)
Thrombocytopenia	13 (17.5)	0 (0.0)
Neutropenia	12 (16.2)	2 (2.7)
Creatinine elevation	9 (12.2)	0 (0.0)
Anemia	6 (8.1)	0 (0.0)

Values are presented as number (%) of patients who reported an adverse event. Percentages may not sum to exactly 100 as patients may have experienced more than one adverse event.

^†^Listed are adverse events and laboratory abnormalities that were reported in at least 5% of patients.

^§^Adverse events were graded according to the National Cancer Institute Common Terminology Criteria for Adverse Events version 4.0

ALT, alanine aminotransferase; AST, aspartate aminotransferase

## Discussion

The present study evaluated the effectiveness and safety of osimertinib treatment after progression on TKI therapy in Chinese patients with advanced/metastatic EGFR T790M-positive NSCLC in a real-world setting. In this cohort of patients, >80% of whom had three or more lines of prior therapy, most (62.1%) showed a partial response to osimertinib treatment, with RR of 67.5% and DCR of 79.8%. The median PFS was 9.0 months and median OS was 12.0 months. The majority of adverse events were low grade (i.e. grades 1–2). The most common adverse events of any grade were nausea (45.9%), diarrhea (31.0%), and decreased appetite (28.3%).

The patients in this real-world study participated in the named-patient program in Macau, which provided an opportunity for them to have early access to osimertinib before it was launched in Macau or nearby countries. Overall, the patients in this study were generally more heavily pre-treated than those in the pivotal osimertinib trials. More than 80% of patients had received at least three prior lines of treatment. In comparison, 68% of patients from the AURA2 trial [4] had previously been treated with at least three lines of anticancer therapies, while the majority of patients (96%) from the AURA3 trial [15] had only previously received one line of treatment. Patients in our study also had worse performance statuses than those in previous osimertinib studies [4]. At the time of osimertinib initiation, some patients in our study were already at the terminal stage and we observed that they had shorter PFS. The median PFS observed in our study (9.0 months) was slightly shorter than those observed in the AURA2 (9.9 months) [4] and AURA3 trials (10.1 months) [15].

Despite these differences, RR in our study was similar to that in published trials (approximately 70%). Consistent with published trials as shown in Table 4 [15–23], the majority of patients (62.1%) in our study showed a partial response to osimertinib. It may also be worth highlighting that the proportion of patients who achieved a complete response in our study (5.4%) was slightly higher than those in the AURA2 and 3 trials (3% and 1%, respectively). Nonetheless, the proportion of patients with progressive disease was higher in our study (20.2%) compared with those in the AURA2 and 3 trials (7% and 6%, respectively), thereby contributing to the lower DCR overall (79.8% vs. AURA2: 92% [4]; AURA3: 93% [15]).

**Table 4:** Real world data of efficacy of osimertinib in pretreated patients with advanced non-small cell lung cancer harboring EGFR T790M mutation.

Author	Population/Study	Number of patients	Age	Female	Asia ethnicity (%)	PFS (months)	OS (months)
Auliac JB[16]	European	205	69.6	68.8%	7.2%	12.6	17.5
Wu YL[17]	ASTRIS study	3014	62	69%	69%	11	1year OS 75.8%
Oh DK[18]	Korea	23	59	56%	100%	7.4	NR
Ahn MJ[19]	AURA and AURA 2 study	411	63	68%	60%	11.2	28.3
Hirashima, T[20]	Japan cohort from AURA and AURA 2	81	NA	NA	100%	13.8	NA
Mok TS[15]	AURA 3 study arm	279	62	62%	65%	10.1	NA
Auliac JB[21]	European population	43	84.6	90.7%	NA	17.5	22.8
Zhou CC[22]	AURA 17 study	171	60	68%	100%	9.7	23.2
Eide, I.J.[23]	TERM study	108	NA	NA	NA	10.8	NA

NR: not reach; NA: not available

It will be interesting to consider these observations for OS (median 12.0 months) alongside the final OS data for AURA3 when they become available. Some differences may be expected because of differences in patient populations and treatment setting; moreover, in both the AURA2 and AURA3 studies, patients were permitted to continue receiving osimertinib beyond disease progression if some clinical benefit was observed.

CNS metastases are common in NSCLC and are challenging to treat because standard chemotherapy do not penetrate the blood-brain barrier [24]. In the present study, a quarter of patients (25.6%) had brain metastases. This group of patients had a shorter median OS than patients without brain metastases (8.0 months vs. 13.0 months, respectively). This observation is interesting in light of preclinical data [25] and clinical studies [4, 15, 26] on the efficacy of osimertinib in patients with NSCLC who had CNS metastases. In the AURA3 study assessing a subgroup of patients with brain metastases [27], the median PFS was 8.5 months for patients with CNS metastases, and 10.8 months for patients without brain metastases. Our results are thus consistent with available data on osimertinib activity in patients with brain metastases.

Higher rates of nausea, decreased appetite, thrombocytopenia, neutropenia, and alanine and aspartate aminotransferase elevation, but lower rates of rash and diarrhea were reported in our study than in the AURA3 study [15]. The observed AEs in our study appear to be consistent with the AE profile of osimertinib detailed in the European Medicines Agency (EMA) package insert [28].

Osimertinib received United States Food and Drug Administration (FDA) [3] and EMA approval [29] in 2018 for first-line treatment of NSCLC patients with EGFR exon 19 deletions or exon 21 L858R mutations, based on the benefit demonstrated in the FLAURA trial [30]. In the FLAURA trial, the median PFS with osimertinib (18.9 months) was significantly longer than that with standard EGFR TKIs (10.2 months) in the overall patient population. This trend was reflected in patients with or without CNS metastases, who had significantly longer median PFS with osimertinib (15.2 months and 19.1 months, respectively) than with standard EGFR TKIs (9.6 months and 10.9 months, respectively). Although OS data were immature at the interim analysis, survival rates at 18 months were comparable between osimertinib and standard EGFR TKIs (83% vs. 71%, respectively). These results expand the range of first-line options for patients with EGFR-mutated NSCLC. Osimertinib is also being evaluated in combination with other agents such as MEK1/2 inhibitors (selumetinib) [31], antiangiogenic agents or anti-EGFR monoclonal antibodies [32], and Bcl-2 inhibitors (navitoclax) [33].

Although this was a relatively small real-world study conducted in a limited population of Chinese patients in the context of a named-patient program, the major findings are consistent with those from pivotal trials of osimertinib as a targeted NSCLC therapy. With recent EMA and FDA approvals in the first-line setting, osimertinib may be a reasonable option for Chinese patients with EGFR T790M-positive NSCLC, with or without progression on TKI or other treatments. In future real-world studies, it will be of interest to address issues such as CNS response, EGFR mutation profile and sequence of therapies, and intrinsic/acquired osimertinib resistance.
